# Predictors of Exacerbations in Chronic Obstructive Pulmonary Disease - Results from the Bergen COPD Cohort Study

**DOI:** 10.1371/journal.pone.0109721

**Published:** 2014-10-03

**Authors:** Gunnar R. Husebø, Per S. Bakke, Marianne Aanerud, Jon A. Hardie, Thor Ueland, Rune Grønseth, Louise J. P. Persson, Pål Aukrust, Tomas M. Eagan

**Affiliations:** 1 Dept. of Thoracic Medicine, Haukeland University Hospital, Bergen, Norway; 2 Dept. of Clinical Science, University of Bergen, Bergen, Norway; 3 Research Institute of Internal Medicine, Oslo University Hospital Rikshospitalet, Oslo, Norway; 4 K.G. Jebsen Inflammatory Research Center, University of Oslo, Oslo, Norway; University of Dundee, United Kingdom

## Abstract

**Background:**

COPD exacerbations accelerate disease progression.

**Aims:**

To examine if COPD characteristics and systemic inflammatory markers predict the risk for acute COPD exacerbation (AECOPD) frequency and duration.

**Methods:**

403 COPD patients, GOLD stage II-IV, aged 44–76 years were included in the Bergen COPD Cohort Study in 2006/07, and followed for 3 years. Examined baseline predictors were sex, age, body composition, smoking, AECOPD the last year, GOLD stage, Charlson comorbidity score (CCS), hypoxemia (PaO2<8 kPa), cough, use of inhaled steroids, and the inflammatory markers leucocytes, C-reactive protein (CRP), neutrophil gelatinase associated lipocalin (NGAL), soluble tumor necrosis factor receptor 1 (sTNF-R1), and osteoprotegrin (OPG). Negative binomial models with random effects were fitted to estimate the annual incidence rate ratios (IRR). For analysis of AECOPD duration, a generalized estimation equation logistic regression model was fitted, also adjusting for season, time since inclusion and AECOPD severity.

**Results:**

After multivariate adjustment, significant predictors of AECOPD were: female sex [IRR 1.45 (1.14–1.84)], age per 10 year increase [1.23 (1.03–1.47)], >1 AECOPD last year before baseline [1.65 (1.24–2.21)], GOLD III [1.36 (1.07–1.74)], GOLD IV [2.90 (1.98–4.25)], chronic cough [1.64 (1.30–2.06)] and use of inhaled steroids [1.57 (1.21–2.05)]. For AECOPD duration more than three weeks, significant predictors after adjustment were: hypoxemia [0.60 (0.39–0.92)], years since inclusion [1.19 (1.03–1.37)], AECOPD severity; moderate [OR 1.58 (1.14–2.18)] and severe [2.34 (1.58–3.49)], season; winter [1.51 (1.08–2.12)], spring [1.45 (1.02–2.05)] and sTNF-R1 per SD increase [1.16 (1.00–1.35)].

**Conclusion:**

Several COPD characteristics were independent predictors of both AECOPD frequency and duration.

## Introduction

Chronic obstructive pulmonary disease (COPD) is a common illness worldwide, and its prevalence is increasing. The disease is heterogeneous where some patients are more prone to have exacerbations than other, proposed to be representing a phenotype of its own [1]. COPD exacerbations are associated with accelerated worsening of lung function [2], [3], increased disease burden and mortality [4], [5], thus making it important to identify and treat these patients.

So far, the best predictors found for future exacerbations are a history of previous exacerbations and decline in forced expiratory volume in one second (FEV1)[6]–[8], together with direct or indirect indicators of pulmonary hypertension [9], [10]. Inflammatory biomarkers may also be of value, alone or several combined [11]–[15]. Other described predictors are depression [16], gastroesophageal reflux disease (GERD) [17], and quality of life [18].

However, the existing knowledge of markers that could predict exacerbation of COPD is still limited. The inflammatory markers associated with COPD exacerbations found so far are unspecific, where abnormal values can be seen in a range of conditions, thus a continued evaluation of novel markers is warranted. Second, although several studies have linked exacerbations with increased inflammation [19], [20], it is not fully understood whether this is a cause or a consequence, and for this purpose longitudinal studies are needed. Third, another measure of disease burden apart from exacerbation frequency is their duration, for which some associations have been described [21]–[24], but patients with delayed exacerbation recovery remains difficult to identify.

This study aimed to find predictors for COPD exacerbations and exacerbation duration, using longitudinal data from a large cohort study in Western Norway, examining both clinical characteristics and novel systemic inflammatory markers.

## Materials and Methods

### Study population

433 Patients with COPD were included in the Bergen COPD Cohort Study (BCCS) between February 2006 and February 2008. All subjects in the study received written and oral information prior to inclusion and signed informed consent. The study was approved by the Regional Committee for Medical and Health Research Ethics, region West (REC-West), case number 165.08. The patients were aged between 44–76 years at the time of inclusion. All patients had a clinical diagnosis of COPD, and a ratio of forced expiratory volume in one second (FEV1) to forced vital capacity (FVC) <0.7 at least 15 minutes after bronchodilation, FEV1<80% predicted by Norwegian reference values [25], and a smoking history of more than 10 packyears. Exclusion criteria were lung diseases other than COPD, additional active inflammatory disease such as various autoimmune disorders, and having a COPD-exacerbation within 4 weeks prior to inclusion. No patients were using long-term prophylactic macrolides or other antibiotics except one patient who was using a tetracycline for a skin disease. The details of study design, patient selection and data collection have been described previously [26].

### Data collection

Briefly, all patients were examined by a study physician. A physical examination, blood gas sampling, and a clinical interview that included exacerbation history, comorbidities and medication history were undertaken. All patients performed spirometry, before and after bronchodilation with 0.4 mg salbutamol, using a Viasys Masterscope. The patients were categorized into Global Initiative for Chronic Obstructive Lung Disease (GOLD 2007) categories II-IV based on post-bronchodilation FEV1.

Body composition was determined with bioelectrical impedance measurements. Cachexia was defined as a fat free mass index (FFMI) less than 17 kg/m^2^ in men or less than 14 kg/m^2^ in women, which corresponds to the lower 95% confidence limit in a normal population [27]. Obesity was defined as a fat mass index (FMI) of more than 9.3 kg/m^2^ in men or more than 13.5 kg/m^2^ in women [27].

All patients were examined and interviewed by a study physician at the out-patient clinic every 6 months for 3 consecutive years. At each visit, the study physician performed a detailed clinical interview, where all exacerbations were registered.

The exacerbation count was the main outcome in this study and was prospectively registered by the patients. We defined an exacerbation as a worsening of respiratory symptoms for two consecutive days or more. Exacerbations that did not require any change in treatment were defined as mild, those requiring treatment with antibiotics or systemic steroids by the decision of a physician were considered moderate, and those in need for hospital admission were considered severe [28]. Exacerbation duration was patient reported, based entirely on symptomatic recovery. The cut off for late recovery was set at three weeks; there exists no definition on a long lasting exacerbation, our limit for when to normally expect recovery was based on clinical experience.

### Laboratory measurements

Peripheral blood sampling and analyses of total leukocyte (WBC) count, C-reactive protein (CRP), neutrophil gelatinase associated lipocalin (NGAL), soluble tumor necrosis factor receptor-1 (sTNF-R1), and osteoprotegrin (OPG) were performed as previously described [26], [29]. WBC and CRP were chosen as inflammatory markers due to their availability as established indicators of inflammation. NGAL [29], sTNF-R1 [26], and OPG [26] have all been shown in cross-sectional analyses from our cohort to be associated with important COPD disease characteristics including FEV1 and exacerbation frequency.

Arterial blood gas analysis was sampled and examined within 5 minutes with a Radiometer ABL520 analyzer [30]. Hypoxemia was defined as a partial oxygen pressure <8.0 kPa.

### Missing Values

30 patients only participated in the baseline visit. Of the 30 patients, 9 were excluded due to use of oral steroids, 8 died before any follow-up visits were performed, in 2 patients CT scans revealed lung cancer, and finally 11 patients withdrew their consent to participate. Thus, 403 patients were included in the statistical analyses. Information regarding chronic cough and cough with phlegm was missing in 8 and 2 patients, respectively. Plasma-sampling failed in 12 patients, and for 1, 2, and 7 patients we lacked sufficient plasma to measure sTNF-R1, OPG, and NGAL, respectively. Arterial blood gas analysis failed in 37 cases, most commonly a sampling error where the patient did not want puncture.

### Statistical analyses

The exacerbation count distribution was heavily skewed to the right. For the baseline characteristics analysis the exacerbation count was dichotomized into patients with an average exacerbation count of less than 1 per year, and those with 1 or more per year. Mild exacerbations were not included in the exacerbation count analysis, as in concordance with prior studies [6]–[8], and due to suspected under-reporting [31]. Bivariate associations were examined with t-tests or non-parametric tests for continuous variables, and χ-square tests for categorical variables.

Random effects negative binomial regression models conditional on gamma errors were fitted to estimate the incidence rate ratios (IRR) for each potential predictor variable. Correspondingly, a multivariate model was fitted including all the predictor variables from the bivariate analyses except for cough with phlegm, which showed a strong colinearity with chronic cough. The inflammatory markers also showed strong colinearity with each other, and were therefore tested separately added to the main model. To test for possible interactions by sex, all variables that differed statistically by sex at baseline were tested one at a time with an interaction term in the final multivariate model.

In addition, exacerbations were analyzed according to duration, searching for factors associated with recovery time exceeding three weeks. A generalized estimating equation logistic regression model with exchangeable correlation structure was fitted, testing potential predictor variables both separate and multivariate. Stata 12.1 (StataCorp LP, College Station, TX, USA) was used for the statistical analyses.

## Results

350 out of the 403 COPD patients experienced one or more exacerbations during the three years of follow-up. A total of 1696 exacerbations were registered, of which 393 were classified as mild, 933 as moderate, and 370 as severe. Women had more exacerbations than men, the difference consisting of more exacerbations of moderate severity (p = 0.001). The median duration for an exacerbation was 14 days (interquartile range 15 days) .

The baseline characteristics of the study population are presented in Table 1. Patients with a higher exacerbation rate were slightly older, more cachectic or obese, had a higher number of exacerbations before inclusion, had a lower FEV1 in % predicted, and had higher frequencies of chronic cough and cough with phlegm. They were also more frequent users of inhaled corticosteroids (ICS). Of the measured inflammatory markers upon inclusion, only CRP was significantly higher in patients with more frequent exacerbations during follow-up.

**Table 1:** Characteristics of COPD patients according to exacerbation frequency during follow-up.

	Less than 1 exacerbationsper year, n = 231	1 or more exacerbationsper year, n = 172	p-Value*
*Sex, %*			0.45
Women	38.1	41.9	
Men	61.9	58.1	
*Age, Mean (SD)*	62.6 (6.8)	64.3 (6.8)	0.01
*Body Composition, %*			0.05
Normal	61.0	48.8	
Cachectic	25.1	31.4	
Obese	13.9	19.8	
*Smoking, %*			0.08
Ex	52.8	61.6	
Current	47.2	38.4	
*Exacerbations last year prior to inclusion, %*			<0.001
0–1	93.5	70.4	
2+	6.5	29.7	
*GOLD 2007 classification, %*			<0.001
FEV1 50–80%	58.9	33.7	
FEV1 30–50%	37.2	47.7	
FEV1<30%	3.9	18.6	
*Hypoxemia, %*			0.07
PaO2>8 kPa	90.9	84.7	
PaO2<8 kPa	9.1	15.3	
Charlson comorbidity Score, %			0.46
1	60.2	54.1	
2	23.8	23.8	
3	10.0	14.0	
4+	06.jan	8.1	
Chronic cough, %			0.002
No	61.5	45.6	
Yes	38.5	54.4	
*Cough with phlegm, %*			0.02
No	45.9	34.3	
Yes	54.1	65.7	
*Use of inhaled steroids, %*			<0.001
No	39.4	22.1	
Yes	60.6	77.9	
*Inflammatory markers, Median (IQR)*			
Leucocyte count (WBC), x10^9^/l	7.7 (6.3 −9.1)	7.9 (6.6 −9.6)	0.11
C-reactive protein (CRP), ng/ml	3.4 (1.7 −6.8)	4.9 (2.1 −12.6)	0.003
Neutrophil gelatinase lipocalin (NGAL), 10 µg/ml	6.7 (5.1 −9.5)	6.7 (5.2 −9.2)	0.77
Soluble TNF receptor-1 (sTNF-R1), 100 µg/ml	6.8 (5.8 −8.1)	7.1 (5.6 −8.5)	0.43
Osteoprotegrin (OPG), ng/ml	5.5 (3.8 −7.1)	5.9 (4.5 −7.3)	0.10

*χ-square for categorical variables, t-test for means and Kruskal Wallis test for medians

### Factors associated with annual exacerbation rate

Table 2 shows bivariate associations between possible predictor variables and annual exacerbation rate. Age, cachexia, number of exacerbations the year before inclusion, GOLD stage, hypoxemia, cough symptoms, and use of ICS were all associated with a higher IRR. Higher levels of CRP and OPG at baseline, but not levels of WBC, NGAL and sTNF-R1, were predictive of a higher exacerbation rate within the follow-up.

**Table 2:** Bivariate predictors of the annual incidence rate ratio (IRR) of moderate or severe COPD exacerbations, estimated by a random effects negative binomial model.

Baseline explanatory variables	IRR	(95% CI)	p-Value
*Sex*			
Men	1		
Women	1.27	(0.99 −1.63)	0.06
*Age*			
per 10 years increase	1.24	(1.04 −1.48)	0.02
*Body Composition*			
Normal	1		
Cachectic	1.41	(1.07 −1.85)	0.02
Obese	1.23	(0.88 −1.71)	0.23
*Smoking*			
Ex	1		
Current	0.83	(0.65 −1.06)	0.13
*Exacerbations 12 months before inclusion*			
0–1	1		
2+	2.74	(2.08 −3.61)	<0.001
*GOLD 2007 classification*			
FEV1 50–80%	1		
FEV1 30–50%	1.75	(1.38 −2.23)	<0.001
FEV1<30%	3.59	(2.51 −5.13)	<0.001
*Hypoxemia*			
PaO2>8 kPa	1		
PaO2<8 kPa	1.61	(1.11 −2.34)	0.01
*Charlson comorbidity Score*			
1	1		
2	1.07	(0.79 −1.43)	0.67
3	1.21	(0.82 −1.77)	0.33
4+	1.37	(0.86 −2.21)	0.19
*Chronic cough*			
No	1		
Yes	1.73	(1.36 −2.19)	<0.001
*Cough with phlegm*			
No	1		
Yes	1.38	(1.08 −1.77)	0.01
*Use of inhaled steroids*			
No	1		
Yes	2.11	(1.62 −2.74)	<0.001
*Inflammatory markers* *			
Leucocyte count (WBC)	1.13	(1.00 −1.29)	0.05
C-reactive protein (CRP)	1.13	(1.01 −1.26)	0.04
Neutrophil gelatinase lipocalin (NGAL)	1.01	(0.90 −1.13)	0.89
Soluble TNF receptor-1 (sTNF-R1)	1.05	(0.94 −1.18)	0.36
Osteoprotegrin (OPG)	1.13	(1.01 −1.27)	0.04

*Per 1 SD increase of marker value.

The adjusted IRRs are shown in Table 3. Significant predictors of a higher risk for moderate or severe exacerbations were female sex, higher age, a history of frequent exacerbations prior to inclusion, higher GOLD stage, chronic cough and use of ICS. Of all potential interactions between sex and the other variables tested, none were found to be statistically significant.

**Table 3:** Multivariate model of the annual incidence rate ratio (IRR) of moderate or severe COPD exacerbations, estimated by a random effects negative binomial model.

Baseline explanatory variables	IRR	95% CI	p-Value
*Sex*			
Men	1		
Women	1.45	(1.14 −1.84)	0.002
*Age*			
per 10 years increase	1.23	(1.03 −1.47)	0.02
*Body composition*			
Normal	1		
Cachectic	1.19	(0.91 −1.56)	0.22
Obese	1.23	(0.90 −1.69)	0.19
*Smoking*			
Ex	1		
Current	0.93	(0.73–1.19)	0.56
*Exacerbations last year*			
0–1	1		
2+	1.65	(1.24–2.21)	0.001
*GOLD 2007 classification*			
FEV1 50–80%	1		
FEV1 30–50%	1.36	(1.07–1.74)	0.01
FEV1<30%	2.90	(1.98–4.25)	<0.001
*Hypoxemia*			
PaO2>8 kPa	1		
PaO2<8 kPa	1.10	(0.79–1.54)	0.58
*Charlson comorbidity Score*			
1	1		
2	0.97	(0.74–1.27)	0.81
3	0.98	(0.68–1.42)	0.93
4+	0.98	(0.61–1.57)	0.93
*Chronic cough*			
No	1		
Yes	1.64	(1.30–2.06)	<0.001
*Use of inhaled steroids*			
No	1		
Yes	1.57	(1.21–2.05)	0.001
**Inflammatory markers added one each, to the above model** *			
Leucocyte count (WBC)	1.04	(0.93–1.17)	0.49
C-reactive protein (CRP)	1.03	(0.93–1.14)	0.56
Neutrophil gelatinase lipocalin (NGAL)	0.99	(0.89–1.10)	0.85
Soluble TNF receptor-1 (sTNF-R1)	1.03	(0.92–1.16	0.56
Osteoprotegrin (OPG)	0.92	(0.82–1.03)	0.15

*IRR per 1 SD increase of marker value.

Thus, mostly the associations seen in the bivariate analyses were confirmed, except for the inflammatory markers, which were not significant predictors of later exacerbations in the model where a large number of covariables were included.

### Factors associated with exacerbation duration more than three weeks

Table 4 and 5 show bi- and multivariate associations between potential predictor variables and exacerbations lasting more than 3 weeks. Variables with significant bivariate associations were obesity, Charlson comorbidity score (CCS) 2, chronic cough, increasing time since inclusion, increasing exacerbation severity, exacerbations during winter and spring (December-February and March-May), and higher levels of baseline sTNF**-**R1. The same variables remained significant after multivariate adjustment, with the exception of obesity, CCS and chronic cough. The presence of hypoxemia was after adjustment associated with exacerbation duration shorter than three weeks. Age, sex, GOLD-stage, smoking status and use of medication along with the other inflammatory markers did not show a significant association with exacerbation duration.

**Table 4:** Bivariate predictors of copd-exacerbation duration more than three weeks, estimated by a generalized estimation equation logistic regression model.

Baseline explanatory variables	OR	95% CI	p-Value
*Sex*			
Men	1		
Women	1.02	(0.79 −1.31)	0.90
*Age*			
per 10 years increase	0.94	(0.78 −1.13)	0.52
*Body Composition*			
Normal	1		
Cachectic	1.07	(0.81 −1.43)	0.63
Obese	1.43	(1.02 −2.00)	0.04
*Smoking*			
Ex	1		
Current	1.24	(0.96 −1.59)	0.10
*Exacerbations 12 months before inclusion*			
0–1	1		
2+	1.20	(0.91 −1.59)	0.20
*GOLD 2007 classification*			
FEV1 50–80%	1		
FEV1 30–50%	1.11	(0.84 −1.45)	0.47
FEV1<30%	1.05	(0.72 −1.53)	0.79
*Hypoxemia*			
PaO2>8 kPa	1		
PaO2<8 kPa	0.83	(0.57 −1.22)	0.34
*Charlson comorbidity Score*			
1	1		
2	1.51	(1.13 −2.02)	0.005
3	1.26	(0.85 −1.85)	0.25
4+	1.21	(0.77 −1.89)	0.42
*Chronic cough*			
No	1		
Yes	1.43	(1.11 −1.85)	0.005
*Cough with phlegm*			
No	1		
Yes	1.18	(0.91 −1.53)	0.22
*Use of inhaled steroids*			
No	1		
Yes	0.97	(0.72 −1.30)	0.83
*Time since inclusion*			
Per year increase	1.2	(1.05–1.36)	0.006
*Exacerbation severity*			
Mild	1		
Moderate (use of antibiotics or steroids)	1.51	(1.12 −2.01)	0.006
Severe (admission to hospital)	2.25	(1.60 −3.17)	<0.001
*Season*			
Summer	1		
Autumn	1.24	(0.90 −1.71)	0.18
Winter	1.48	(1.09 −2.01)	0.01
Spring	1.36	(0.99 −1.86)	0.06
*Inflammatory markers* *			
Leucocyte count (WBC)	1.11	(0.98 −1.25)	0.10
C-reactive protein (CRP)	0.94	(0.83 −1.07)	0.36
Neutrophil gelatinase lipocalin (NGAL)	1.06	(0.93 −1.21)	0.37
Soluble TNF receptor-1 (sTNF-R1)	1.14	(1.01 −1.28)	0.04
Osteoprotegrin (OPG)	0.97	(0.85 −1.09)	0.59

*IRR per 1 SD increase of marker value.

**Table 5:** Multivariate model of copd-exacerbation duration more than three weeks, estimated by a generalized estimation equation logistic regression model.

Baseline explanatory variables	OR	95% CI	p-Value
*Sex*			
Men	1		
Women	1.17	(0.88–1.56)	0.29
*Age*			
per 10 years increase	0.88	(0.70–1.10)	0.27
*Body Composition*			
Normal	1		
Cachectic	0.92	(0.66–1.29)	0.63
Obese	1.35	(0.93–1.98)	0.11
*Smoking*			
Ex	1		
Current	1.29	(0.95–1.76)	0.11
*GOLD 2007 classification*			
FEV1 50–80%	1		
FEV1 30–50%	1.23	(0.90–1.67)	0.19
FEV1<30%	1.18	(0.75–1.87)	0.48
*Hypoxemia*			
PaO2>8 kPa	1		
PaO2<8 kPa	0.60	(0.39–0.92)	0.02
*Charlson comorbidity Score*			
1	1		
2	1.14	(0.81–1.59)	0.46
3	1.15	(0.72–1.82)	0.56
4+	1.36	(0.76–2.42)	0.30
*Chronic cough*			
No	1		
Yes	1.29	(0.97–1.71)	0.08
*Time since inclusion*			
Per year increase	1.19	(1.03–1.37)	0.02
*Exacerbation severity*			
Mild	1		
Moderate (use of antibiotics or steroids)	1.58	(1.14–2.18	0.006
Severe (admission to hospital)	2.34	(1.58–3.49)	<0.001
*Season*			
Summer	1		
Autumn	1.33	(0.94–1.89)	0.11
Winter	1.51	(1.08–2.12)	0.02
Spring	1.45	(1.02–1.35)	0.04
* Soluble TNF receptor-1 (sTNF-R1)*			
per 1 SD increase of marker value	1.16	(1.00–1.35)	0.05

## Discussion

We used prospective follow-up data from a large cohort study from western Norway to identify predictors for COPD exacerbation frequency and duration. The best predictors of future exacerbations in this study were a history of frequent exacerbations prior to inclusion, lower lung function, increasing age, and female sex, confirming the findings of earlier studies [6]–[8]. Furthermore, we identified some easily accessible clinical variables independently associated with increased exacerbation rates such as chronic cough and the use of inhaled steroids.

Exacerbation duration was significantly associated with exacerbation severity and season, which is in accordance with other studies [21], [22], [24]. In addition, we identified an association between exacerbation duration and both hypoxemia and sTNF-R1 not demonstrated before.

The main strengths of this study were the prospective design, the large number of patients, and the assessment of a series of different variables. This allowed for the use of complex regression analysis and adjustment for several key variables. Attendance rate at the visits was high, varying between 86 and 97 percent. The longitudinal design allowed for predictive statistical modeling, however, no intervention was done, and the concept prediction should not be confused with causality.

The statistical analysis of exacerbation frequency is complex, due to its distorted distribution and due to clustering both in subject and time [32]. One approach is to compare the frequent vs. the non-frequent exacerbator using logistic regression. However, negative binomial or Poisson regression may be more suited [33]. In our study we treated the exacerbation frequency as a count variable. Both Poisson and negative binomial models were considered, but due to overdispersion of the data the latter model was preferred, though a Poisson model was also fitted producing almost identical results (not shown here).

The exacerbation data was acquired through interview by the study physician, with the aid of the patients’ journal present. The majority of patients (n = 350) lived in a proximity to our hospital, which would have led them to attend our hospital in an emergency. Due to the long follow-up, we did not use an exacerbation diary or other grading tools although these methods have been validated [34]. Thus, we believe under-reporting of severe exacerbations were highly unlikely and under-reporting of moderate exacerbations unlikely but probably present to some extent. Regarding severe exacerbations, apart from hospital admission and duration, we had no other clinical information to validate its severity, and due to this we chose to analyze severe and moderate exacerbations together. For the analysis of exacerbation duration, mild exacerbations were included in the model despite the limitations in in the data collection mentioned above, and this should be taken into consideration when interpreting the data.

This study showed a large diversity in both exacerbation frequency and duration. In agreement with earlier studies, a person with exacerbations in the past is more likely to experience exacerbations in the future [6]–[8]. Earlier studies have shown that women experience more symptoms from their illness, but mortality rates have shown gender equality [35]. A similar picture emerged in our study, where women experienced a higher rate of moderate exacerbations, but not severe exacerbations requiring hospital admission. Whether this represents a genuine increase in exacerbations or an increased tendency among women to seek medical attention remains unclear.

Somewhat surprising, but also seen before [6], was the finding that ex-smokers had no reduction of the exacerbation count compared to active smokers. This could imply that smoking cessation was too late, and that disease progression continued after smoking cessation. Another explanation may be that most of our cohort consisted of a selection of COPD patients having had prior consultations with pulmonary physicians [26], which might affect smoking habits, where perhaps the most symptomatic patients were more likely to have quit prior to entering the study. Different exacerbation rates between smokers and ex-smokers have been seen in COPD patients selected from a more general population [36]. Finally, time since smoking cessation was not a significant variable in our study (data not shown), but our study may not have been powered to examine that properly.

The association between exacerbation frequency and use of ICS may seem paradoxical as large randomized studies have shown a modest, but significant decrease in exacerbation risk with their use [37]–[40]. Nevertheless, randomized trials often include highly selected study populations, and non-intervention cohort studies as ours add to the existing knowledge. Several studies have shown an association between ICS and pneumonia rate [41]–[43], and it is possible that use of ICS comes with an increased risk for infectious exacerbations of COPD. We observed an exacerbation IRR of 1.57 in ICS-users after multivariate adjustments, but still we cannot exclude a selection bias since these patients may have received their ICS due to worsening of symptoms not accounted for in our model. Also, in our study, a majority of the patients using ICS (91%) were using them in combination with a long acting beta-2-agonist (LABA), making it difficult to separate the effect of the ICS vs. the LABA. Thus, our observation of increased exacerbation rate in ICS-users cannot be interpreted as a causal effect, but nonetheless, an observation of ICS-use may aid in identifying a patient with increased risk for future exacerbations.

A primary objective in this study was to evaluate the association between systemic inflammation and exacerbation rate measuring inflammatory markers at inclusion. In our study we only measured the inflammatory markers at baseline, and their predictive value for an event up to three years later is likely to decrease as time goes by. CRP and OPG at baseline were bivariately associated with the exacerbation rate, but not after multivariate adjustment. WBC, fibrinogen and uric acid have independently shown predictive value in other studies [6], [11], [14], indicating that systemic inflammation may be a prerequisite for exacerbations. CRP has shown predictive value in combination with the WBC and fibrinogen [12], and the specificity and sensitivity may be further improved with the construction of so called inflammomas [15], containing three or more easily accessible markers, or with sputum samples, nonetheless these approaches have still yielded limited clinical value, so the search for additional markers should continue.

It is challenging to predict those patients in risk for a long lasting exacerbation. Dissimilar from the exacerbation rate, factors like prior exacerbations and FEV1 did not seem to affect the duration. Increasing values of sTNF-R1 as a marker of activity in the TNF system was associated with late exacerbation recovery, and may be a marker of chronic inflammation in COPD. On the other hand, sTNF-R1 is associated with important comorbidities difficult to adjust for [26], and this finding must be confirmed in other studies. The association between duration and season can be linked to both lower temperature [44] and seasonality of viral infections [45], and was anticipated; on the other hand, the observation of reduced recovery time in patients with hypoxemia was unexpected. Hypoxemic patients are likely to have more symptoms in their stable state, perhaps making it difficult to distinguish between the exacerbated and the stable state, which might affect their exacerbation reporting. Nonetheless, this observation should be confirmed in other studies.

This study illustrates the vicious circle affecting a large proportion of COPD patients, where one exacerbation predisposes for the next, leading to an ever increasing disease burden. This underscore the need for markers that could further identify patients at risk, as well as the need for proper intervention in these patients. We identify several clinical parameters for recognition of patients at risk for frequent or long lasting exacerbations, making it possible for earlier or more extensive preventive intervention. Finally, there is still a lack of useful inflammatory markers, both to identify patients with high risk of future exacerbations, as well as a diagnostic tool to detect ongoing exacerbations.
